# Cortical Bone Thickness, Base Osteophyte Occurrence and Radiological Signs of Osteoarthritis in the Fingers of Male Elite Sport Climbers: A Cross-Sectional 10-Year Follow-Up Study

**DOI:** 10.3389/fphys.2022.893369

**Published:** 2022-06-02

**Authors:** Torsten Pastor, Stefan Fröhlich, Tatjana Pastor, Jörg Spörri, Andreas Schweizer

**Affiliations:** ^1^ Department of Orthopaedic and Trauma Surgery, Cantonal Hospital Lucerne, Lucerne, Switzerland; ^2^ Division of Hand Surgery, Department of Orthopaedics and Trauma Surgery, Balgrist University Hospital, University of Zurich, Zurich, Switzerland; ^3^ University Center for Prevention and Sports Medicine, Department of Orthopaedics, Balgrist University Hospital, University Zurich, Zurich, Switzerland; ^4^ Sports Medical Research Group, Department of Orthopaedics, Balgrist University Hospital, University of Zurich, Zurich, Switzerland; ^5^ Department of Plastic and Hand Surgery, Inselspital University Hospital Bern, University of Bern, Bern, Switzerland

**Keywords:** climbing, degeneration, overuse, finger degeneration, osteophyte, load adaption

## Abstract

**Background:** Sport climbing places high mechanical loads on fingers. In 2012, our research group demonstrated adaptations of climbers’ cortical bones with the presence of osteophytes compared to non-climbing controls.

**Objectives:** 1) To investigate 10-year changes in cortical bone thickness, base osteophyte occurrence and radiological signs of osteoarthritis in the fingers of elite male sport climbers with more than 25 years of climbing history and 2) to compare cortical bone thickness, base osteophyte occurrence and radiological signs of osteoarthritis between male sport climbers and age-matched controls at the 10-year follow-up.

**Methods:** All 31 elite sport climbers who participated in both the baseline and 10-year follow-up assessments (follow-up rate 100%) were examined by means of X-rays. Cortical bone thickness, presence of osteophytes and signs of osteoarthritis according to Kellgren-Lawrence were obtained and compared to the baseline values 10 years earlier and to age-matched controls at the follow-up (*n* = 15).

**Results:** Significantly increased cortical bone thickness over the past 10 years was observed in climbers (mean absolute difference with 95% CI:0.98 mm (0.77 mm, 1.19 mm); *p* <0.001). Moreover, compared to age-matched controls, climbers had significantly thicker cortical bone at the 10-year follow-up (mean absolute difference with 95% CI:0.86 mm (0.61 mm, 1.12 mm); *p* <0.001). In climbers, osteophytes and clear signs of osteoarthritis were mainly seen in DIP joints. Signs of osteoarthritis according to Kellgren-Lawrence were more prevalent than 10 years before in most joints. In lateral radiographs, base osteophytes were not significantly more prevalent than 10 years before in most of the joints. The percentage of climbers who had osteophytes in any DIP (PIP) joint increased from 93.5% (67.7%) at baseline to 100% (74.2%) at the 10-year follow-up. The percentage of climbers who had clear signs of osteoarthritis according to Kellgren-Lawrence in any DIP (PIP) joint increased from 12.9% (9.7%) at baseline to 74.2% (64.5%) at 10-year follow-up. Only a few such degenerative changes were found in age-matched controls.

**Conclusion:** An accumulation of repetitive climbing-related stress to the fingers of elite sport climbers over the career may induce lifelong mechano-adaptation of the cortical bone thickness of all phalanges. At the 10-year follow-up, a further significant increase in radiographic signs of osteoarthritic changes was observed.

## Introduction

With inclusion in the Olympic program for the 2020 Tokyo Summer Games, sport climbing continues to become a popular and fast-growing sport (Lutter et al., 2017). However, research in this area is still about to develop, and the long-term impact of intensive sport climbing to the human body is relatively unknown, as previous research mainly focused on acute climbing-related injuries and performance. Climbing requires holding the entire body weight with sometimes only one finger, resulting in extreme forces on the bones and connective tissue (Moor et al., 2009). Cortical adaptations in long-term climbers and a correlation with their years of climbing have already been demonstrated (Bollen and Wright, 1994; Rohrbough et al., 1998; Morrison and Schoffl, 2007; Schoffl et al., 2007; Allenspach et al., 2011; Hahn et al., 2012; Schoffl et al., 2015; Schoffl et al., 2018).

Ten years ago, our research group investigated the influence of high mechanical stress from climbing on the fingers of 31 elite level sport climbers and demonstrated a remarkably high occurrence of osteophytes and thicker cartilage in PIP and DIP joints compared to age-matched controls (Allenspach et al., 2011; Pastor et al., 2020). Other authors have also reported osteoarthritis-like changes in the fingers of long-time climbers; although slightly different populations were investigated and other assessment criteria were applied, likely leading to different occurrence frequencies (Bollen and Wright, 1994; Schoffl et al., 2018). However, whether these findings are early signs of osteoarthritis or just mechano-adaptation could not be conclusively clarified at that time. Furthermore, it is unclear how these adaptations evolve over a long observation period in elite sport climbers compared to non-climbing controls.

Therefore, the aims of this study were to investigate the 10-year changes in cortical bone thickness, base osteophyte occurrence and radiological signs of osteoarthritis in the fingers of male sport climbers with more than 15 years of climbing history at baseline and 25 years at follow-up and to compare these parameters between the climbers at the 10-year follow-up and age-matched controls.

## Methods

### Study Design and Participants

Ten years after baseline assessments (Allenspach et al., 2011; Hahn et al., 2012), all 31 elite rock climbers were reinvestigated as part of follow-up assessments. At the reinvestigation, climbers were aged 48.3 ± 5.0 years on average (Table 1 *for detailed characteristics*). Inclusion criteria in addition to participation in the two baseline studies were rock climbing on the elite level (minimum 7b + on the French scale) and a minimum of 25 years of climbing (range of climbing experience: 25–42 years; mean: 32 years). At the time of the baseline examination, climbers were at a level of 7b + to 9a + (average 8b +), and at the time of the follow-up, climbers were at a level of 6b to 9a (average 7c +). Exclusion criteria were major operations or injuries to the hands, quitting climbing activities or rejected informed consent; however, none of the climbers had to be excluded. All participants were contacted over a time period from April to August 2019 by telephone and could be examined 10 years after both baseline studies (follow-up rate of 100%).

**TABLE 1:** Characteristics of the climbers at the 10-year follow-up.

Variable	Mean ± SD	95%CI
Age (y)	48.3 ± 5.0	(46.5, 50.0)
Body Weight (kg)	72.8 ± 7.2	(70.3, 75.3)
Body Height (m)	1.78 ± 0.04	(1.76, 1.79)
Body Mass Index (kg/m^2^)	23.0 ± 2.3	(22.2, 23.8)
Years of Climbing (y)	31.6 ± 4.4	(30.1, 33.2)
Average Weekly Climbing Hours (h)	13.9 ± 7.3	(11.4,16.4)

SD, standard deviation; CI, confidence interval.

In contrast to the climbers, the age-matched controls investigated 10 years earlier were unfortunately not available for re-examination in the current study due to the fact that we either had no current contact data, they had moved, or they were unwilling to participate after being re-contacted by phone and/or email. Therefore, 15 new non-climbing participants (mean age 48.1 ± 6.1 years) from different occupational fields at Balgrist University Hospital were recruited through personal inquiry and served as sex- and age-matched controls. In addition to not participating in any climbing activities or regularly performing physically demanding tasks, the eligibility criteria were the same as those for the climbers. This study was approved by the local ethics committee (Cantonal Ethics Commission Zurich, Switzerland, BASEC-Nr. 2019–00677), and all participants signed a written informed consent form.

### Data Collection and Evaluation

**FIGURE 1:**
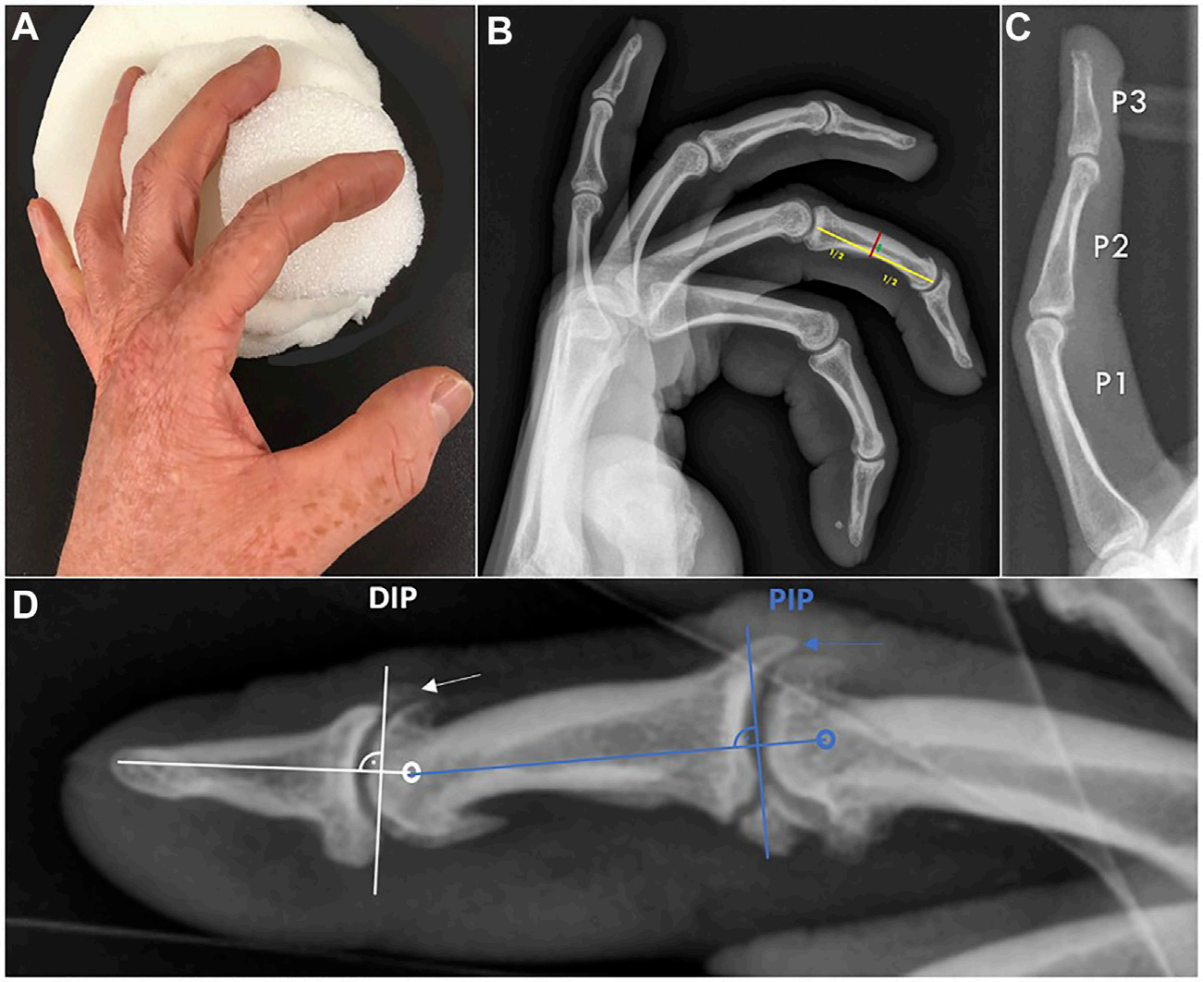
**(A)** To obtain standardized lateral radiographs of all fingers, a custom-made device was used. **(B)** Climbers: lateral radiograph of a left hand using this device. Note how all fingers are projected strictly laterally and every single cortex is visible. Measurement of the intermediate phalanx of digit III is demonstrated as an example. Red line: outer cortical width. Green line: inner cortical width. Yellow line: length of the phalanx. **(C)** Controls: exemplary depicted lateral radiograph of a left middle finger. Note the clear differences in cortical thickness and medullary canal width in contrast to the climber. P1: proximal phalanx, P2: intermediate phalanx, P3: distal phalanx. **(D)** Lateral standardized radiograph of a left digit II in a climber. Modified measurement principles according to Allenspach et al. are demonstrated. Dorsal base osteophytes were rated as present or absent at the DIP and PIP joints, respectively. The arrows mark the presence of osteophytes at the DIP and PIP joints.

All participants received standardized anterior-posterior and lateral X-ray views (Ysio wi-D system, Siemens, Erlangen, Germany) of all fingers except the thumb of both hands using a custom-made positioning devise to ensure standardized lateral X-ray images (Figure 1A). The same device was used in both previously conducted baseline studies (Allenspach et al., 2011; Hahn et al., 2012). No blinding of the investigators was applied. For each phalanx of all fingers except the thumb, two measurements were obtained digitally in the lateral view (Figure 1B) according to Bollen and Wright (Bollen and Wright, 1994). After the length of the phalanx was determined, the inner cortical width and the outer cortical width were measured exactly in the middle of the phalanx as previously done in the baseline investigation (Hahn et al., 2012). With these two parameters, cortical bone thickness was determined as the difference between outer cortical width and medullary width. Cortical bone thickness was only evaluated in the lateral view due to the more pronounced differences compared to non-climbers in the baseline investigation (Hahn et al., 2012).

Base osteophyte occurrence was evaluated in lateral radiographs according to Allenspach et al. (Allenspach et al., 2011). Due to the most pronounced osteophyte occurrence on the dorsal base of the phalanx of PIP and DIP joints in the baseline investigation, only these osteophytes were rated as present or absent in the current study as follows (Figure 1D): a line was laid through the centre of rotation of the joint and along the axis of the related finger bone in the distal direction. Afterwards, a line was drawn perpendicular to this line adjacent to the socket. Osteophytes in DIP and PIP joints were rated as present if they reached the vertical line or if they were already broken.

Radiological signs of osteoarthritis (OA) were rated on antero-posterior radiographs according to the Kellgren-Lawrence (K-L) classification (Kellgren and Lawrence, 1957). Kellgren and Lawrence developed the score in 1957, which is used to classify the severity of osteoarthritis based on conventional radiographs and was later accepted by the World Health Organization (WHO) as the radiological definition of OA for the purpose of epidemiological studies (Sangha, 2000; Schiphof et al., 2008). The following signs of osteoarthritis are considered: evidence of osteophytes, decrease in joint space width (cartilage thickness), increased subchondral sclerosis and deformity of the joint-forming bone parts (osteophytes). PIP and DIP joints were rated to one of the following grades:

grade 0 (none): definite absence of X-ray changes of osteoarthritis;

grade 1 (minimal): doubtful joint space narrowing and possible osteophytic lipping;

grade 2 (moderate): definite osteophytes and possible joint space narrowing;

grade 3 (severe): moderate multiple osteophytes, definite narrowing of joint space, sclerosis and deformity of bone ends.

According to Kellgren and Lawrence, osteoarthritis is deemed present at grade 2 (Kellgren and Lawrence, 1957; Rohrbough et al., 1998; Schoffl et al., 2018).

### Statistical Analysis

Statistical analysis included the following steps: 1) Cortical bone thickness from the lateral view at phalanx 1–3 [proximal (P1), intermediate (P2), distal (P3)] of both hands and digits II-V was reported as the mean ± SD; 2) corresponding cortical bone thickness differences and interaction effects were tested by the use of a repeated-measures multivariate ANOVA (*p* <0.05). Within-subject factors were phalanx [proximal (P1), intermediate (P2), distal (P3)], side (right and left) and digit (Dig II-V), and between-subject factor was the group (climbers at baseline, climbers at 10 years follow-up, and age-matched controls). Effect sizes were reported as partial eta^2^, and following Cohen (Cohen, 1988), effect size thresholds were taken as 0.01 (small effect), 0.06 (medium effect), and 0.14 (large effect); 3) detailed group differences for each joint/side/digit were tested using unpaired sample t-tests backed up by bias-corrected accelerated bootstrapping with 1,000 samples (*p* <0.05); and 4) for all groups, the relative frequency of base osteophyte occurrence, as well as of subjects with K-L scores of 2 or higher (='clear' signs of OA), was plotted as the percentage proportion (number affected subjects/number of subjects per group × 100) with corresponding 95% CI. Non-overlapping 95% CIs between the groups were interpreted as significant differences at *p* <0.05.

## Results

### Differences in Bone Thickness

The descriptive statistics of cortical bone thickness from the lateral view for the 3 groups (climbers at baseline, climbers at 10-year follow-up and age-matched non-climbing controls), phalanx (proximal (P1), intermediate (P2) and distal (P3), sides (right and left), and digits (Dig II-V) are presented in Table 2. Climbers demonstrated thicker cortical bones than age-matched controls as well as 10 years earlier. The exact statistical results of the repeated-measures multivariate ANOVA were as follows: There were significant differences and large effects in cortical bone thickness between the groups (*p* <0.001; partial eta^2^ = 0.667), phalanx (*p* <0.001; partial eta^2^ = 0.945), and digits (*p* <0.001; partial eta^2^ = 0.862) on the multivariate level. Interaction effects revealed for phalanx*group (*p* <0.001, partial eta^2^ = 0.661), side*group (*p* = 0.001, partial eta^2^ = 0.185), digit*group (*p* <0.001, partial eta^2^ = 0.348), phalanx*digit (*p* <0.001, partial eta^2^ = 0.406) and phalanx*digit*group (*p* <0.001, partial eta^2^ = 0.224). Significantly increased cortical bone thickness over the past 10 years was observed in the climbers (mean absolute difference with 95% CI: 0.98 mm (0.77 mm, 1.19 mm); *p* <0.001). Moreover, compared to age-matched controls, climbers had significantly thicker cortical bone at the 10-year follow-up (mean absolute difference with 95% CI: 0.86 mm (0.61 mm, 1.12 mm); *p* <0.001). In part, non-significant differences in cortical bone thickness existed between climbers at baseline and at the 10-year follow-up at the distal phalanx (P3).

**TABLE 2:** Descriptive and inferential statistics for the bone thickness at phalanx 1–3 of both hands and digits II-V for climbers at baseline and at 10 years follow-up, as well as their age-matched controls.

Structure	Climbers at baseline (A)^a^		Climbers at 10-years follow-up (B)		Age-matched Controls (C)		Pairwise comparisons (t-tests^b^)			
	*n*	Mean ± SD	*n*	Mean ± SD	*n*	Mean ± SD	B-A (95%CI)	*p* value	C-B (95%CI)	*p* value
Right hand	—	—	—	—	—	—	—	—	—	—
P1 D2 (mm)	31	4.3 ± 0.6	31	5.6 ± 0.5	15	5.0 ± 0.6	1.3 (1.0, 1.5)	0.001***	0.6 (0.3, 1.0)	0.002**
P2 D2 (mm)	31	3.2 ± 0.5	31	4.7 ± 0.5	15	3.8 ± 0.5	1.5 (1.2, 1.7)	0.001***	0.9 (0.6, 1.2)	0.001***
P3 D2 (mm)	31	3.0 ± 0.5	31	3.0 ± 0.4	15	2.4 ± 0.4	0.1 (-0.1, 0.3)	0.499^ns^	0.7 (0.4, 0.9)	0.001***
P1 D3 (mm)	31	4.5 ± 0.5	31	6.2 ± 0.8	15	5.2 ± 0.6	1.7 (1.3, 2.0)	0.001***	1.0 (0.6, 1.5)	0.001***
P2 D3 (mm)	31	3.3 ± 0.5	31	4.9 ± 0.7	15	3.9 ± 0.6	1.6 (1.3, 1.9)	0.001***	0.9 (0.5, 1.3)	0.001***
P3 D3 (mm)	31	3.1 ± 0.5	31	3.2 ± 0.5	15	2.5 ± 0.4	0.1 (-0.2, 0.4)	0.308^ns^	0.7 (0.4, 1.0)	0.001***
P1 D4 (mm)	31	3.9 ± 0.5	31	5.1 ± 0.6	15	4.5 ± 0.5	1.3 (1.0, 1.5)	0.001***	0.6 (0.3, 0.9)	0.002**
P2 D4 (mm)	31	2.8 ± 0.4	31	4.5 ± 0.6	15	3.5 ± 0.5	1.7 (1.4, 1.9)	0.001***	1.0 (0.7, 1.3)	0.001***
P3 D4 (mm)	31	2.8 ± 0.4	31	3.1 ± 0.7	15	2.2 ± 0.4	0.3 (-0.1, 0.6)	0.135^ns^	0.9 (0.6, 1.2)	0.001***
P1 D5 (mm)	31	3.2 ± 0.4	31	4.0 ± 0.5	15	3.5 ± 0.7	0.8 (0.6, 1.1)	0.001***	0.5 (0.1, 0.9)	0.009**
P2 D5 (mm)	1	2.8 ± 0.4	31	3.3 ± 0.5	15	2.7 ± 0.5	0.5 (0.3, 0.8)	0.001***	0.7 (0.4, 0.9)	0.001***
P3 D5 (mm)	31	2.3 ± 0.3	31	2.5 ± 0.4	15	1.8 ± 0.3	0.2 (0.0, 0.4)	0.022*	0.7 (0.5, 0.9)	0.001***
Left hand	—	—	—	—	—	—	—	—	—	—
P1 D2 (mm)	31	4.4 ± 0.5	31	5.8 ± 0.8	15	4.9 ± 0.7	1.4 (1.1, 1.7)	0.001***	1.0 (0.6, 1.4)	0.001***
P2 D2 (mm)	31	3.2 ± 0.5	31	4.5 ± 0.6	15	3.7 ± 0.4	1.4 (1.1, 1.7)	0.001***	0.9 (0.6, 1.1)	0.001***
P3 D2 (mm)	31	2.9 ± 0.5	31	3.0 ± 0.5	15	2.1 ± 0.3	0.1 (-0.2, 0.3)	0.426^ns^	1.0 (0.7, 1.2)	0.001***
P1 D3 (mm)	31	4.5 ± 0.5	31	6.4 ± 0.7	15	5.2 ± 0.6	1.9 (1.6, 2.2)	0.001***	1.2 (0.8, 1.6)	0.001***
P2 D3 (mm)	31	3.2 ± 0.5	31	5.1 ± 0.7	15	3.8 ± 0.6	1.9 (1.6, 2.2)	0.001***	1.3 (0.9, 1.6)	0.001***
P3 D3 (mm)	31	3.1 ± 0.6	31	3.2 ± 0.6	15	2.3 ± 0.4	0.1 (-0.1, 0.4)	0.371^ns^	0.9 (0.6, 1.2)	0.001***
P1 D4 (mm)	31	3.8 ± 0.5	31	5.4 ± 0.7	15	4.2 ± 0.5	1.6 (1.2, 1.9)	0.001***	1.1 (0.8, 1.5)	0.001***
P2 D4 (mm)	31	2.8 ± 0.5	31	4.5 ± 0.7	15	3.5 ± 0.5	1.7 (1.4, 2.0)	0.001***	1.1 (0.7, 1.4)	0.001***
P3 D4 (mm)	31	2.7 ± 0.4	31	3.4 ± 1.2	15	2.0 ± 0.4	0.7 (0.3, 1.2)	0.037*	1.4 (1.0, 1.9)	0.006**
P1 D5 (mm)	31	3.2 ± 0.4	31	3.9 ± 0.6	15	3.6 ± 0.8	0.8 (0.5, 1.0)	0.001***	0.4 (-0.1, 0.8)	0.113^ns^
P2 D5 (mm)	31	2.7 ± 0.4	31	3.2 ± 0.5	15	2.7 ± 0.4	0.5 (0.3, 0.7)	0.001***	0.5 (0.2, 0.7)	0.001***
P3 D5 (mm)	31	2.2 ± 0.3	31	2.6 ± 0.4	15	1.9 ± 0.4	0.4 (0.2, 0.6)	0.001***	0.8 (0.6, 1.0)	0.001***

a Data already partially presented in Hahn et al. (2012). The climbers at the 10-year follow-up (B) were the same subjects as the climbers at baseline (A).

b Level of significance-based t-tests and backed up by bias-corrected accelerated bootstrapping with 10,000 samples: ^ns^, not significant, *p <0.05, **p <0.01, ***p <0.001.

P1, proximal phalanx; P2, intermediate phalanx; P3, distal phalanx; D2, Dig II; D3, Dig III; D4, Dig IV; D5, Dig V; n, number of observations.

### Differences in Base Osteophyte Occurrence

**FIGURE 2:**
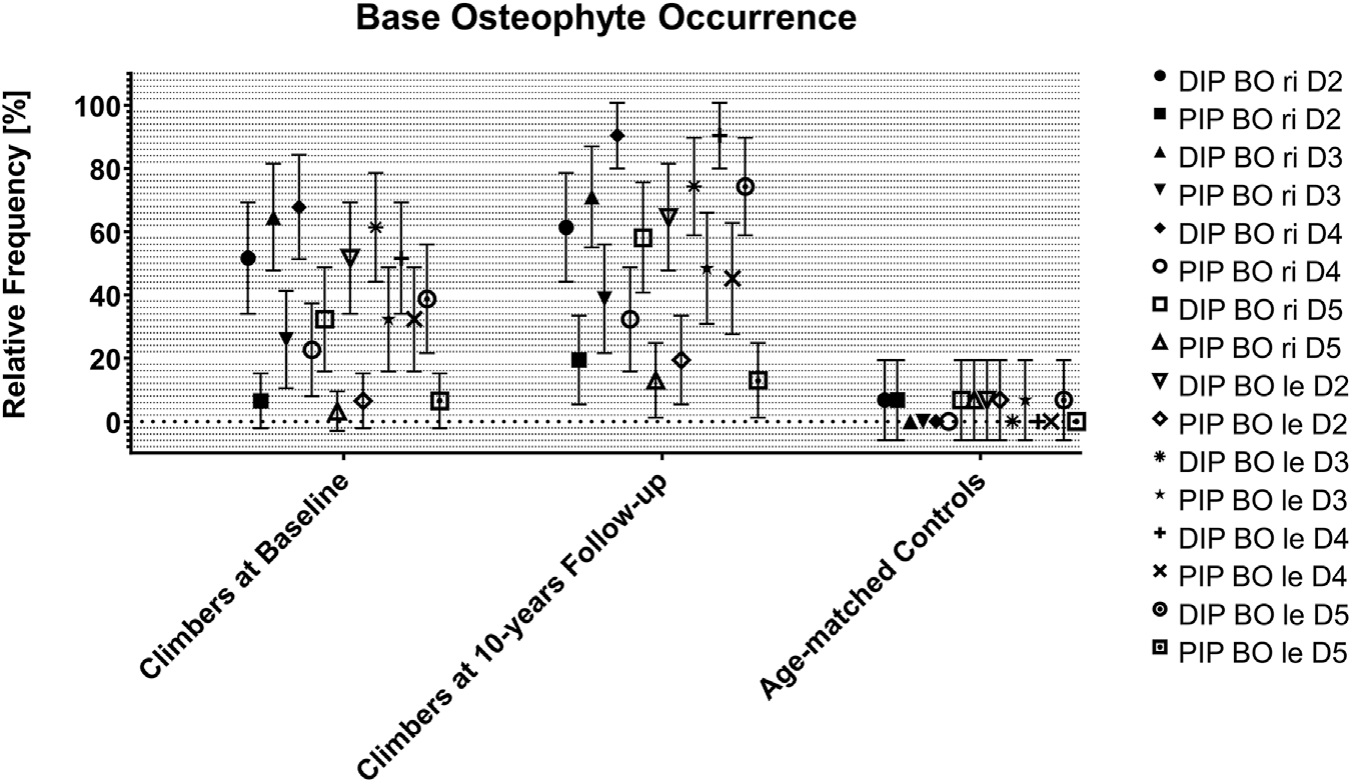
Base osteophyte occurrence in climbers at baseline and at 10 years follow-up, as well as in their age-matched controls. Data are expressed as joint, side and digit-specific relative proportion group means with 95% CI. BO: base osteophyte; DIP: distal interphalangeal joint; PIP: proximal interphalangeal joint; ri: right; le: left; D: digit.

Figure 2 and Supplementary Figures S1A–D show the relative proportions of osteophyte occurrence at the base in climbers at baseline and after 10 years and in age-matched controls.

The age matched controls at the 10-year follow-up showed relative frequencies of base osteophytes between 0 and 10% in all joints of all fingers. The climbers at the 10-year follow-up showed relative frequencies higher than 60% in all DIP joints and between 10 and 50% in all PIP joints. Thus, mainly the DIP joints were affected by base osteophytes, most severely on Dig IV, followed by Dig III and Dig V.

In all DIP joints of all fingers, the relative occurrence of base osteophytes showed a significant difference between the climbers at the 10-year follow-up and the non-climbing controls. In PIP joints, this difference was only seen in Dig III (both sides), Dig IV (both sides), and Dig V (left side only).

The comparison between climbers at baseline and climbers at the 10-year follow-up revealed significant differences only for the DIP joints of the left Dig IV and V, while all other joints showed no significant differences. However, the relative frequency of base osteophytes slightly increased in all other joints over the 10-year follow-up period without reaching statistical significance (Supplementary Figures S1A–D). The percentage of climbers who had osteophytes in any DIP joint increased from 93.5% at baseline to 100% at the 10-year follow-up (non-climbing controls: 13.3%). For any PIP joint, this percentage increased from 67.7 to 74.2% (non-climbing controls: 13.3%).

### Differences in ‘Clear’ Signs of OA

**FIGURE 3:**
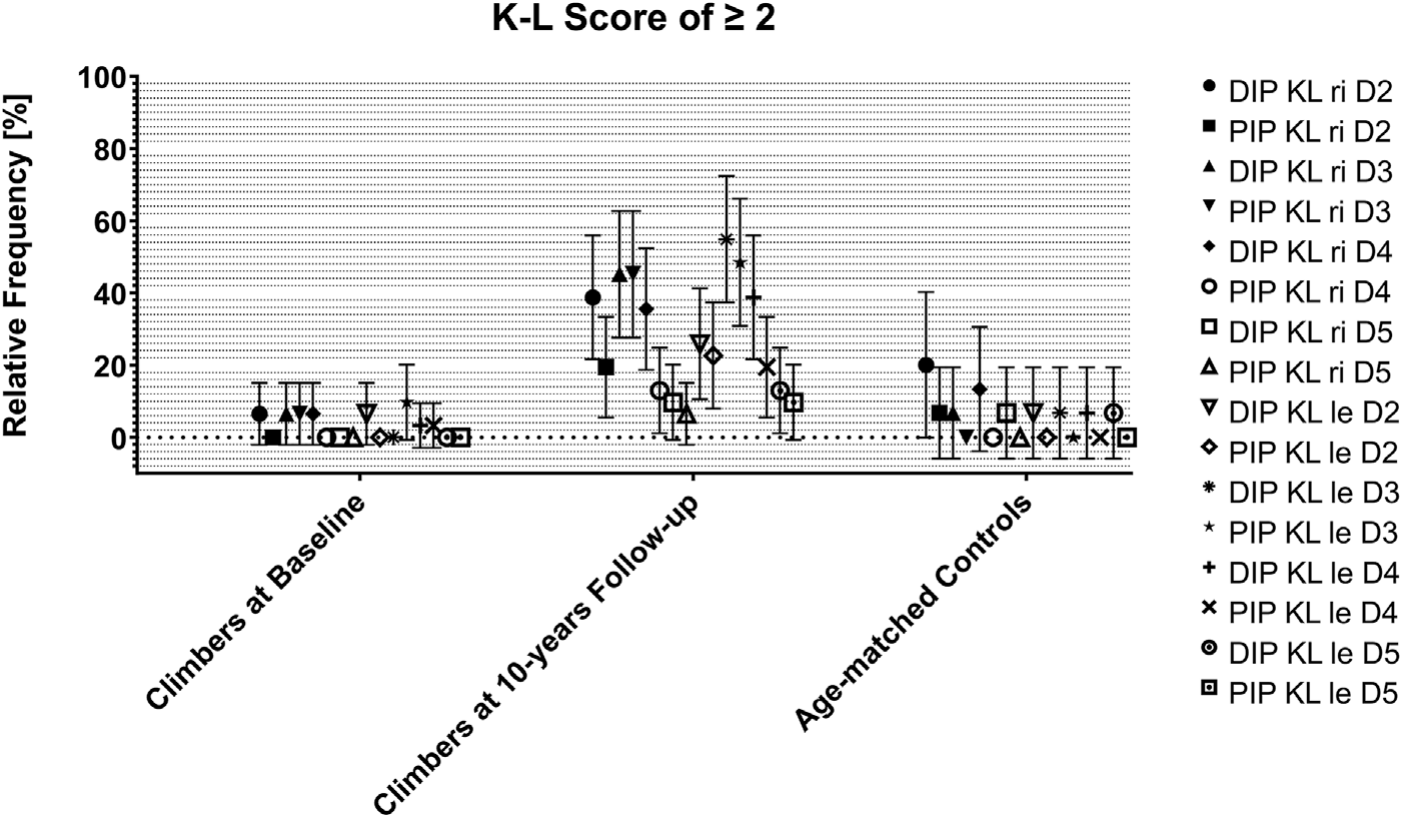
Occurrence of ‘clear’ signs of OA (= K-L scores of 2 or higher) in climbers at baseline and at the 10-year follow-up, as well as in their age-matched controls. Data are expressed as joint, side and digit-specific relative proportion group means with 95% CI. KL: Kellgren-Lawrence Score; DIP: distal interphalangeal joint; PIP: proximal interphalangeal joint; ri: right; le: left; D: digit.

**FIGURE 4:**
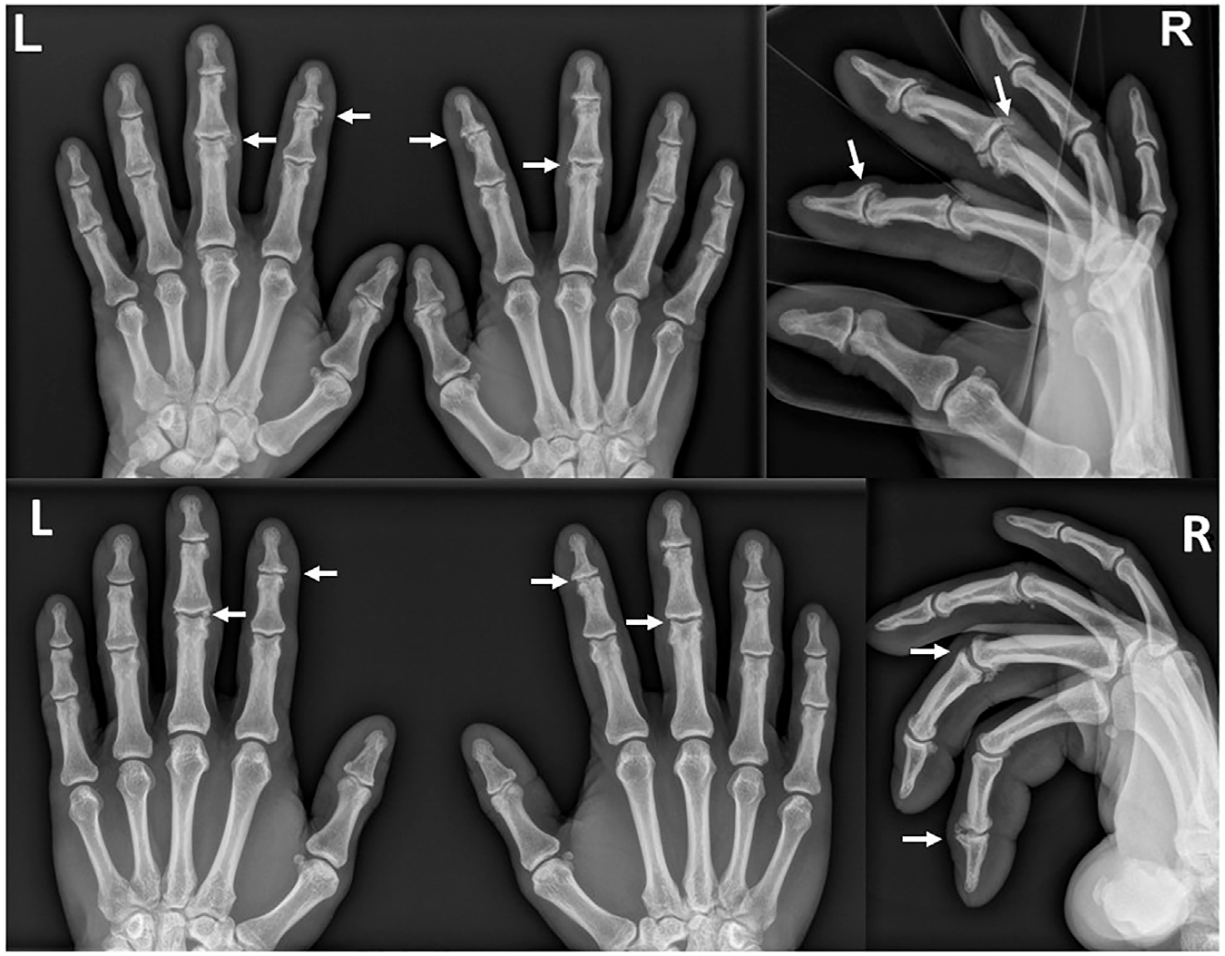
Anteroposterior (left) and lateral (right) radiographs of the same climber: top, current images; bottom, images at baseline 10 years earlier. Note the increased signs of osteoarthritis with larger osteophytes and decreased joint spaces in the current images in contrast to the baseline examination. Particularly impressive findings are marked with arrows as an example.

Figure 3, Figure 4 and Supplementary Figures S2A–D show the relative proportions of subjects with K-L scores of 2 or higher (‘clear signs of OA) in climbers at baseline and after 10 years and in age-matched controls.

Climbers at baseline and non-climbing controls both showed relative frequencies of clear signs of osteoarthritis in all joints of all fingers between 0 and 20%. Climbers at the 10-year follow-up showed relative frequencies between 5 and 60%, depending on the joint and finger. Clear signs of osteoarthritis in terms of a Kellgren-Lawrence score of 2 or higher were most frequently seen in the DIP joints of several fingers. The DIP joints of Dig III were affected most frequently, and the PIP joints of Dig V were affected least frequently.

Compared to the climbers at baseline 10 years before, the relative frequency of clear signs of osteoarthritis was significantly increased in the right DIP joint of Dig II, in both PIP joints of Dig II, in both DIP joints and both PIP joints of Dig III, in both DIP joints and the right PIP joint of Dig IV, and in the left DIP joint of Dig V. Climbers at the 10-year follow-up presented significantly more frequently with clear signs of osteoarthritis in the left PIP joint of Dig II, in both DIP joints and both PIP joints of Dig III, and in the left DIP joint and both PIP joints of Dig IV than non-climbing controls (Supplementary Figures S2A–D). The percentage of climbers who had clear signs of osteoarthritis in any DIP joint increased from 12.9% at baseline to 74.2% at the 10-year follow-up (non-climbing controls: 26.7%). For any PIP joint, this percentage increased from 9.7 to 64.5% (non-climbing controls: 6.7%).

## Discussion

The main findings of the current study, which is the study with the longest climbing history (25–41 years) of its participants, were as follows: 1) in climbers, a significant increase in cortical bone thickness over the last 10 years was observed; 2) at the 10-year follow-up, cortical bone thickness was still significantly larger in climbers than in age-matched, non-climbing controls; 3) in climbers, the frequency of the occurrence of base osteophytes (in lateral radiographs) has not significantly increased during the 10-year observation period in most joints; 4) in contrast to the base osteophytes, the frequency of clear signs of osteoarthritis in ap-radiographs has significantly increased over the 10 years in most joints; 5) in climbers, DIP joints are more frequently affected by both base osteophytes and osteoarthritis than PIP joints; 6) base osteophytes and osteoarthritis are significantly more frequent in climbers than in age-matched controls in many, but not all finger joints; 7) while base osteophytes in the lateral view in climbers are most pronounced in Dig IV, clear signs of osteoarthritis in the ap-view are most pronounced in Dig III.

### Mechano-Adaptation of Cortical Bone Thickness

In the baseline investigation 10 years earlier, thicker cortical bones in all phalanges of elite sport climbers compared to age-matched non-climbing controls have been reported (Hahn et al., 2012). The current study revealed a further increase in cortical thickness in all phalanges of the same elite sport climbers. This is in line with previously published reports regarding mechano-adaptation of fingers in sport climbers. Bollen et al. and Schöffl et al. reported cortical reactions to stress in the fingers of elite sport climbers (Bollen and Wright, 1994; Schoffl et al., 2004; Schoffl et al., 2007; Schoffl et al., 2018). However, only the study by Schöffl et al. was a longitudinal study, with a similar follow-up time of 11 years, but with significantly younger participants (Schoffl et al., 2018). In addition to mechano-adaptation of bones, several studies have demonstrated adaptations in other structures of the fingers of elite sports climbers. Schreiber et al. reported adaptations with thicker palmar plates, pulleys and flexor tendons compared to a non-climbing control group (Schreiber et al., 2015). In a 10-year follow-up study of the same climbers, all investigated soft tissue parameters were thicker compared to the baseline investigation, which suggests a theory of a life-long build-up of these soft tissue structures (Fröhlich et al., 2021). Similar findings were also reported by Rohrbough et al. (Rohrbough et al., 1998), Garcia et al. (Garcia et al., 2018) and Klauser et al. (Klauser et al., 2000). Furthermore, thicker capsules and collateral ligaments were reported in 20 sport climbers compared to an age-matched control group (Heuck et al., 1992). Thus, the findings of the current study suggest a career-long build-up of cortical bone thickness in the fingers of elite sport climbers.

### Development of Osteophytes and Osteoarthritis in the Fingers of Elite Sport Climbers

In contrast to cortical bone thickness, the occurrence of base osteophytes did not significantly increase in most joints of the climbers over the 10-year observation period. A possible explanation is the fact that all climbers already had at least 15 years of intensive climbing experience at baseline (Allenspach et al., 2011). Therefore, the authors suggest that the formation of base osteophytes in high-level climbers occurs primarily during the first 15 years of the climbing career and progresses less than other structural adaptations thereafter (i.e., the upcoming 10 years). This seems to be true at least for the question of whether an osteophyte is present or not, regardless of its extent.

With regard to clear signs of osteoarthritis in the ap-view, these are significantly more frequent in most joints of the climbers than 10 years earlier. The baseline study of climbers with more than 15 years of climbing experience has already shown that osteophytes can be seen early in lateral radiographs, while ap radiographs can still look relatively normal (Allenspach et al., 2011). The new findings (now 10 years later in climbers with more than 25 years of climbing experience) indicate that previously already present osteophytes have increased to such an extent that they may be considered as visible signs of osteoarthritis in the ap-view.

The finding that DIP joints are more affected by degenerative changes than PIP joints is novel, as previous studies have reported a similar occurrence of osteophytes in both proximal and distal interphalangeal joints (Allenspach et al., 2011; Pastor et al., 2020). This finding suggests that at later stages of the career, the DIP joints seem to be more prone to degeneration than the PIP joints. This might be explained by the long-term effect of the particularly high loads on the DIP joints, for example, when applying the “crimp position”, which also served as an explanation for the particularly pronounced thickening of the palmar plates in the DIP joints of climbers (Schreiber et al., 2015). A consequence could be the recommendation that the crimp position should no longer be trained in excess at an older age. However, the current study is not fully able to justify this recommendation and more research is needed.

While osteophytes and signs of osteoarthritis are significantly more frequent in climbers than in age-matched controls in most finger joints, this is still not the case for all joints. This may allow two conclusions to be drawn: on the one hand, it can be assumed that even after many years of intensive climbing, certain finger joints are still subjected to significantly less stress than others: the joints least prone to osteophyte formation and other signs of osteoarthritis are PIP at Dig V and PIP at Dig II. On the other hand, the age-matched control subjects of the current study are now also 10 years older than those of the baseline studies, and with correspondingly increasing age, it can be assumed that generally more joints show certain degenerations, which may lead to a convergence between climbers and non-climbers.

The finding that osteophytes in the lateral view are most common in the DIP joints of Dig IV is in line with the theory that this finger must withstand the greatest mechanical forces, which has been stated in previous studies (Roloff et al., 2006; Schoffl and Schoffl, 2007; Vigouroux et al., 2008; Schoffl et al., 2018). However, we found most of the clear signs of osteoarthritis in the ap-view in the DIP joints of Dig III. Other studies have also found both fingers, Dig III and IV, to be frequently subject to degeneration (Allenspach et al., 2011; Pastor et al., 2020). Therefore, we recommend considering both fingers as particularly affected by degenerative changes in climbers.

### Methodological Considerations

Although a follow-up rate of 100% over 10 years was achieved and it is worth mentioning that the climbers assessed in the current study represent the study sample with the longest climbing history (25–41 years) at the elite level reported in the literature to date, some possible limitations should be considered when interpreting the study results.

First, the sample size was identical to the population selected 10 years ago, which is why only a limited number of 31 elite male climbers were examined. As a direct consequence, the generalizability of the current findings for other cohorts may be limited and with regard to conclusions for clinical practice, some caution is advised. With respect to the reported significant results, potential type I errors of statistical testing (the risk of rejecting the null hypothesis when it's actually true, i.e., concluding that there is a difference between groups when in fact such difference does not exist) may have occurred. In addition, with regard to the reported nonsignificant results, there could be potential type II errors of statistical testing (i.e., the risk of accepting the null hypothesis when it's actually false, i.e., concluding that there is no difference between groups when in fact such a difference exists).

Second, radiographic measurements were performed by two different examiners 10 years ago and now. Although all examinations were highly standardized and easy to perform, interobserver bias still may have been possible. Furthermore, all examinations were performed using the same technical devices and protocols to minimize discrepancies, and the senior author was involved in both studies.

Third, the non-climbing controls of 15 men examined in the baseline investigation could not be recruited again for the current study; therefore, a new age-matched control group was recruited. In addition, the recruitment of the control group by personally enquiring employees at our hospitals may have led to some selection bias. However, this potential bias was counteracted by selecting subjects from different health care professions, but who mainly worked in an office setting (i.e., did not perform physically demanding tasks). Finally, examiners have not been blinded to the climbing status, as the hand radiographs of elite climbers are usually immediately recognizable.

## Conclusion

Climbing at the elite level likely induces mechano-adaptation of cortical bones in the fingers, and build-up takes place over the career. Climbers show higher frequencies of base osteophytes in PIP and DIP joints of most fingers compared to controls; however, it does not significantly increase over the later 10 years of the career in most fingers. In contrast, clear radiographic signs of osteoarthritis also increase at later stages of the climbing career (i.e., more than 15 years of climbing), especially in DIP joints and Dig III and IV. These results were obtained from a population of climbers with the longest climbing experience compared to the literature (mean 32 years).

## Data Availability

The original contributions presented in the study are included in the article/Supplementary Material, further inquiries can be directed to the corresponding author.
