# Supplementary material for: Cortical Bone Thickness, Base Osteophyte Occurrence and Radiological Signs of Osteoarthritis in the Fingers of Male Elite Sport Climbers: A Cross-Sectional 10-Year Follow-Up Study
Source: Front Physiol. 2022 Jun 2;13:893369. doi: 10.3389/fphys.2022.893369 (PMC9203125; doi:10.3389/fphys.2022.893369)
Supplement: Supplementary file 1 [file Image1.pdf]

Supplementary Material

1.1 Supplementary Figures

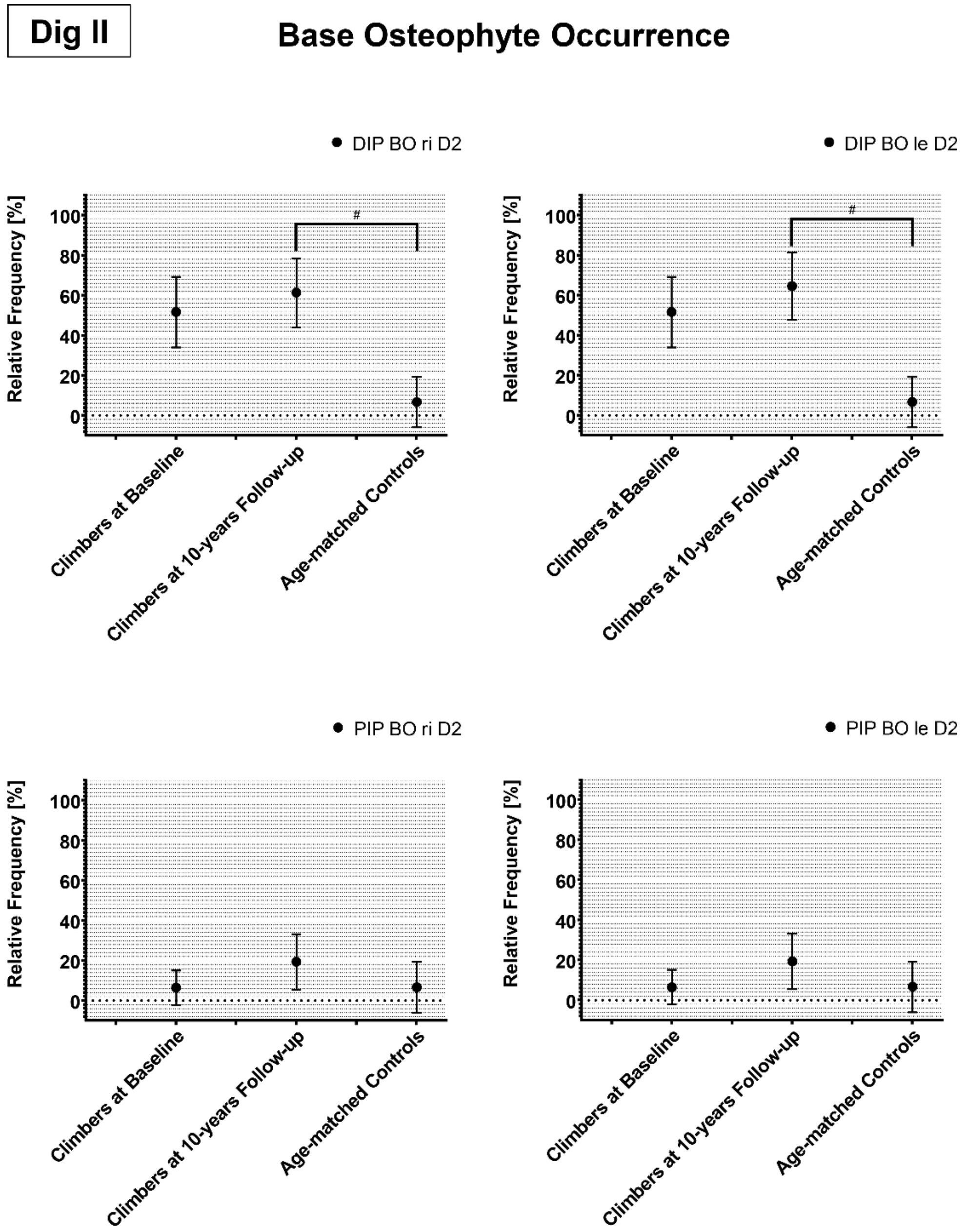

Supplemental Figure 1a: Base osteophyte occurrence in climbers at baseline and at 10 years follow-up, as well as in their age-matched controls for digit II. Data are expressed as joint and side-specific relative proportion group means with 95% CI. Non-overlapping 95% CIs between the groups were interpreted as significant differences at $p<0.05$; # climbers at 10-years follow-up significantly differ from age matched controls.

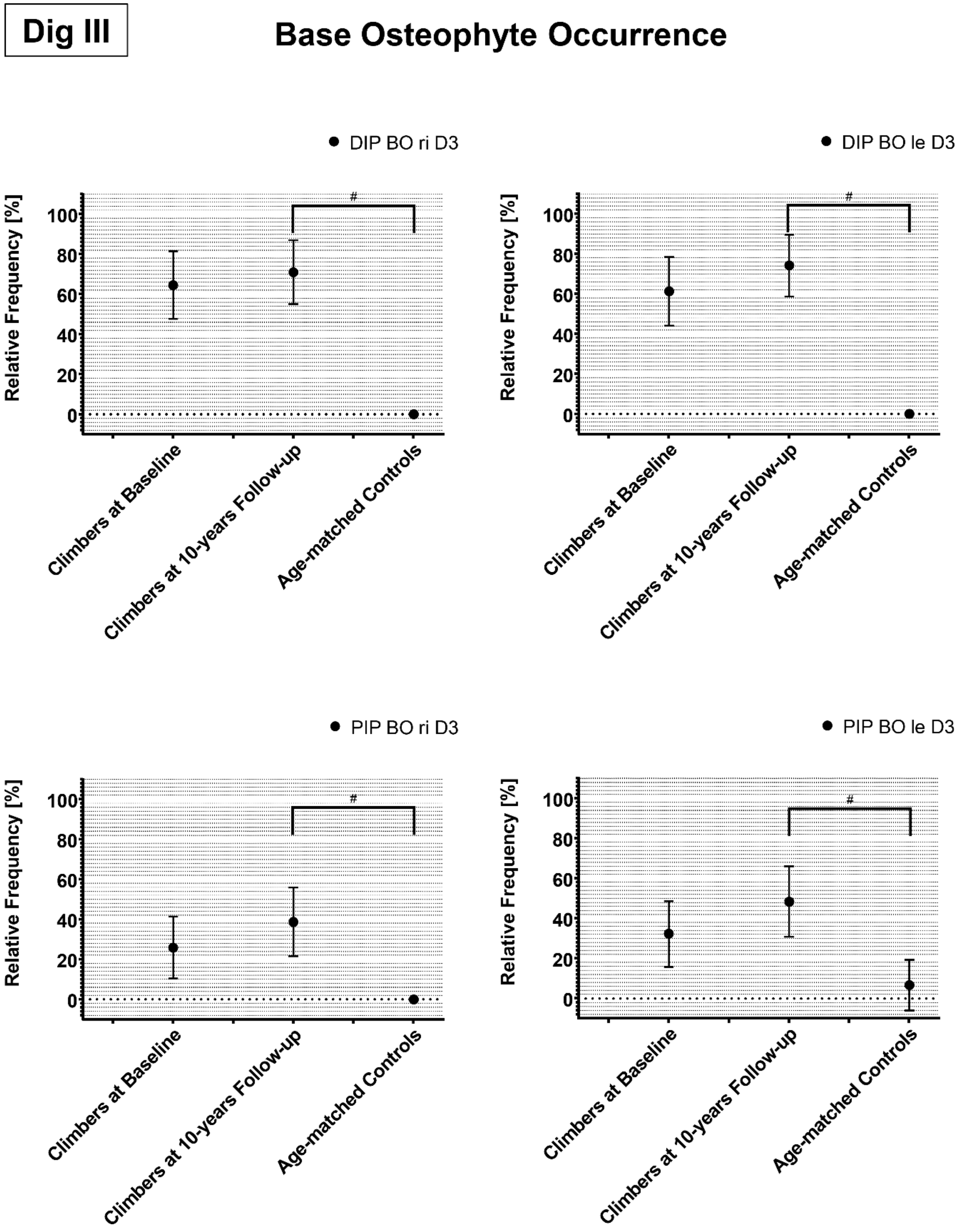

Supplemental Figure 1b: Base osteophyte occurrence in climbers at baseline and at 10 years follow-up, as well as in their age-matched controls for digit III. Data are expressed as joint and side-specific relative proportion group means with 95% CI. Non-overlapping 95% CIs between the groups were interpreted as significant differences at $p<0.05$; # climbers at 10-years follow-up significantly differ from age matched controls.

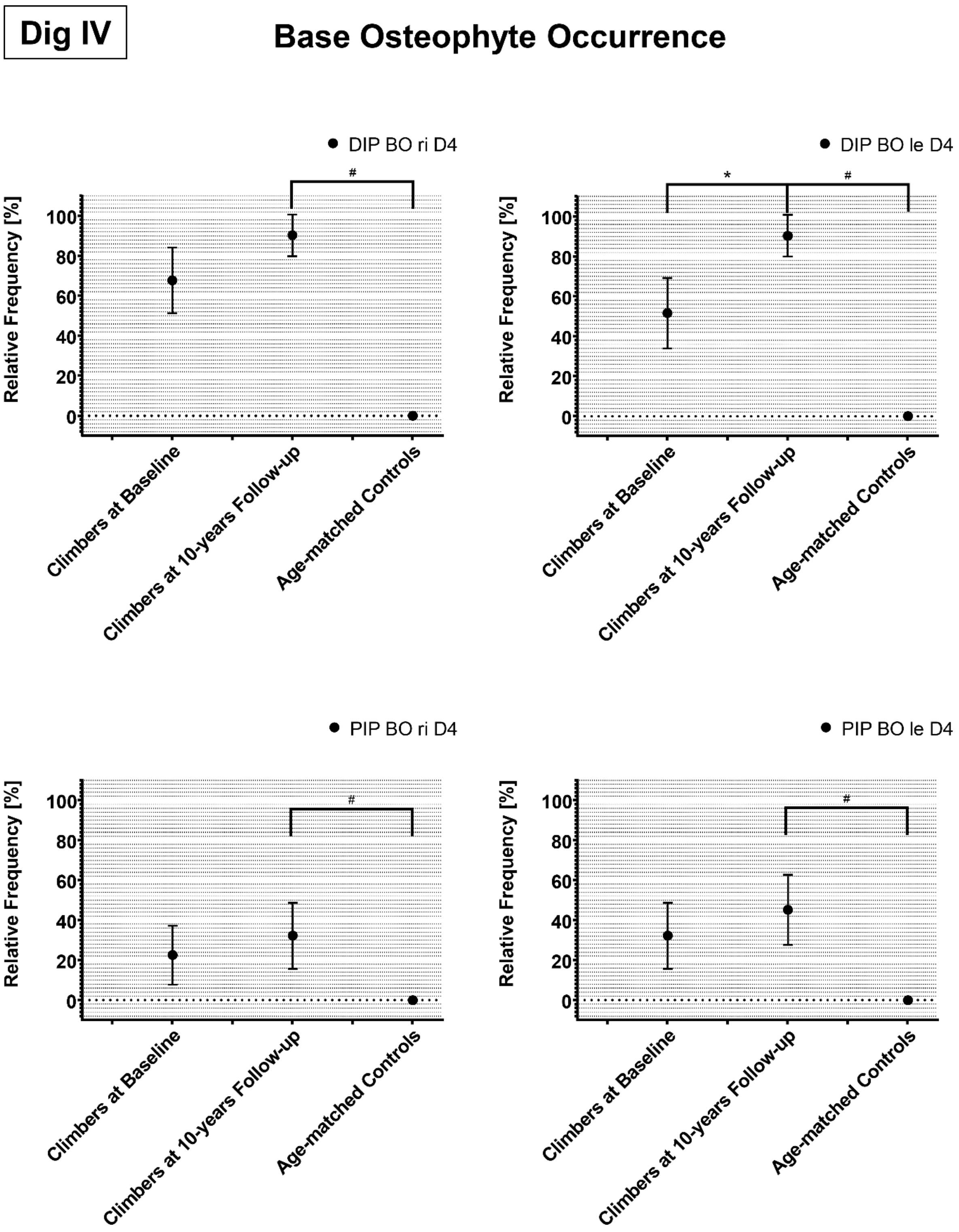

Supplemental Figure 1c: Base osteophyte occurrence in climbers at baseline and at 10 years follow-up, as well as in their age-matched controls for digit IV. Data are expressed as joint and side-specific relative proportion group means with 95% CI. Non-overlapping 95% CIs between the groups were interpreted as significant differences at $p<0.05$; * climbers at baseline significantly differ from climbers at 10-years follow-up; # climbers at 10-years follow-up significantly differ from age matched controls.

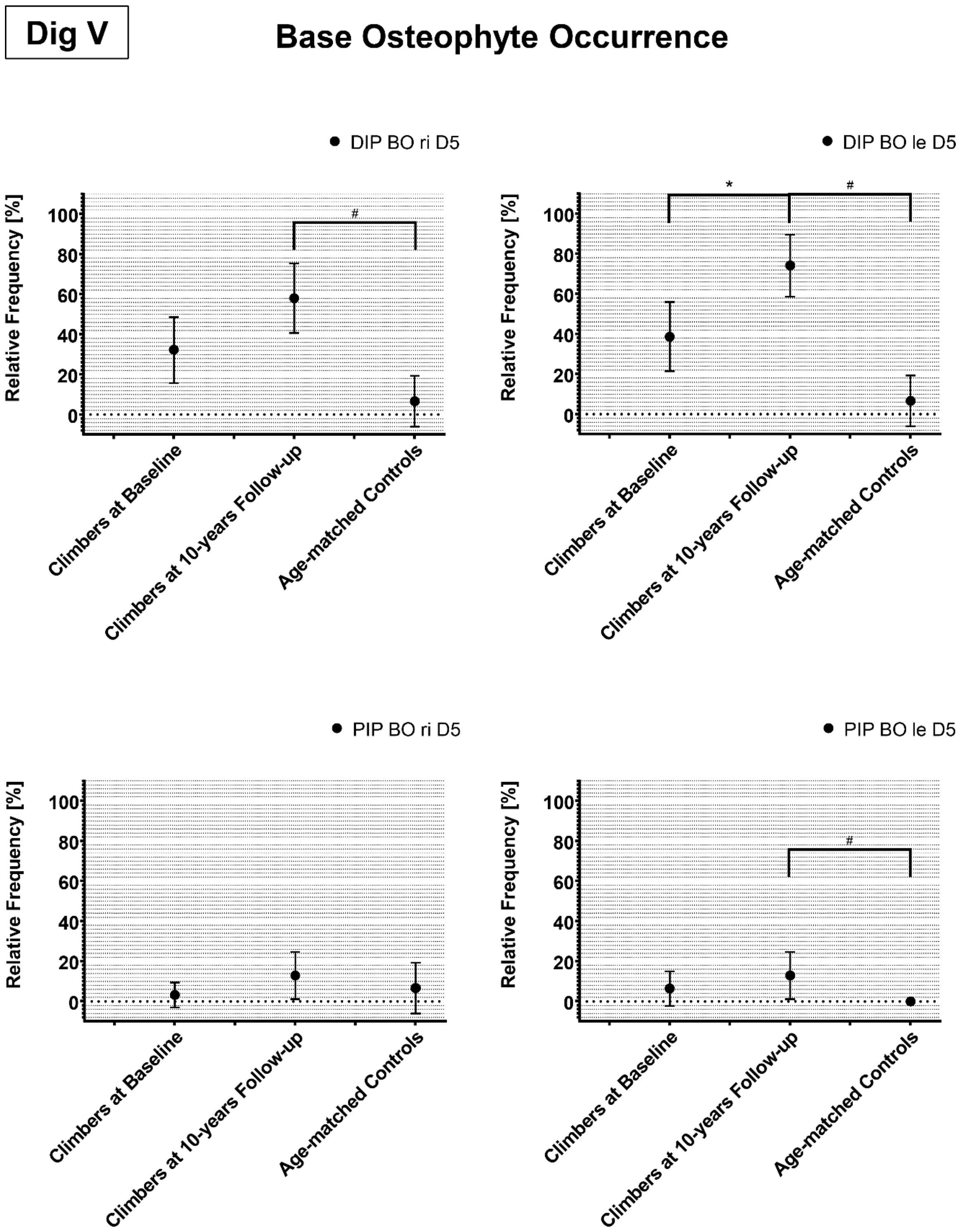

Supplemental Figure 1d: Base osteophyte occurrence in climbers at baseline and at 10 years follow-up, as well as in their age-matched controls for digit V. Data are expressed as joint and side-specific relative proportion group means with 95% CI. Non-overlapping 95% CIs between the groups were interpreted as significant differences at $p<0.05$; * climbers at baseline significantly differ from climbers at 10-years follow-up; # climbers at 10-years follow-up significantly differ from age matched controls.

Dig II

K-L Score of ≥ 2

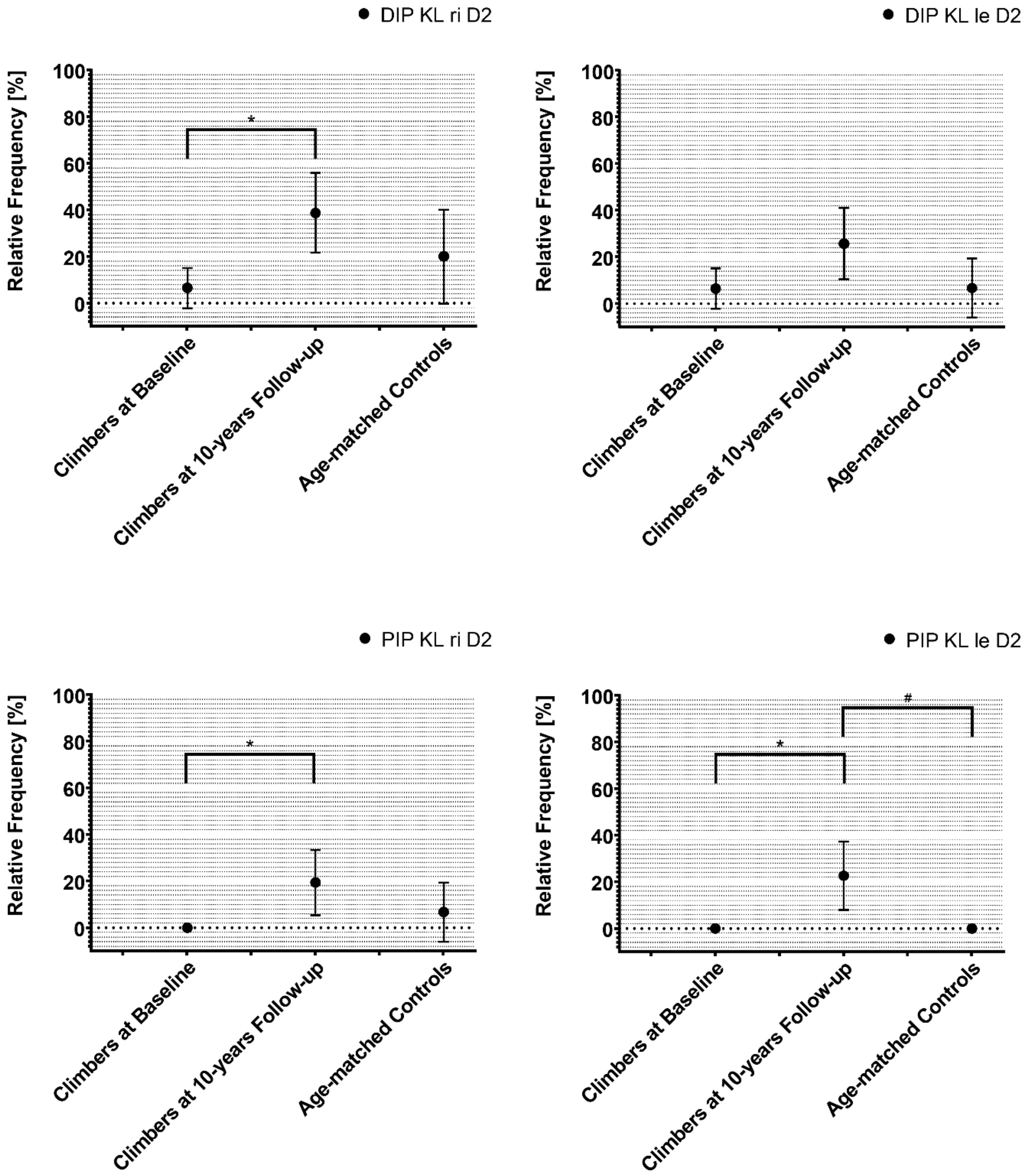

Supplemental Figure 2a: Occurrence of 'clear' sings of OA (= K-L scores of 2 or higher) in climbers at baseline and at 10 years follow-up, as well as in their age-matched controls for digit II. Data are expressed as joint and side-specific group relative proportion means with 95% CI. Non-overlapping 95% CIs between the groups were interpreted as significant differences at $p<0.05$; * climbers at baseline significantly differ from climbers at 10-years follow-up; # climbers at 10-years follow-up significantly differ from age matched controls.

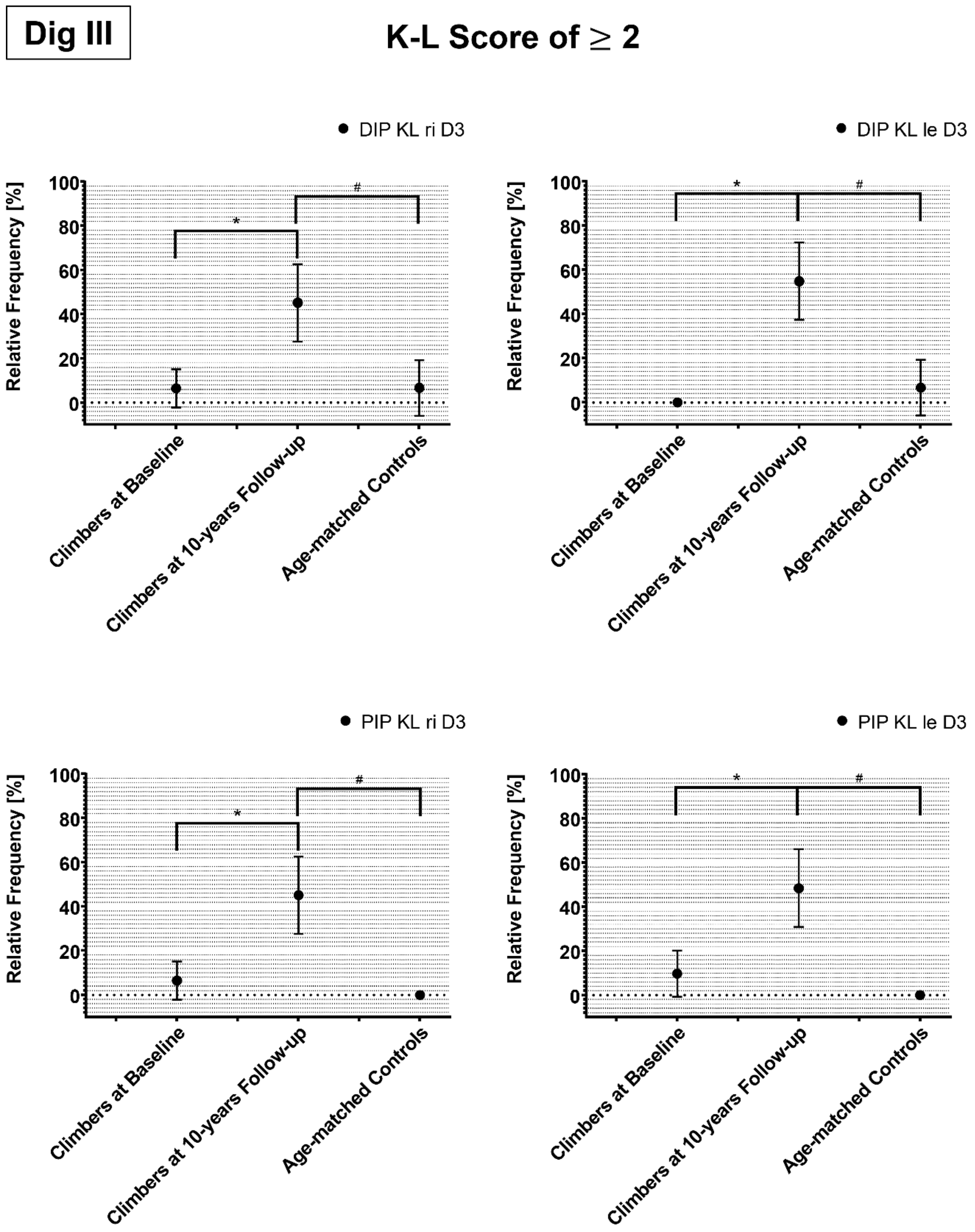

Supplemental Figure 2b: Occurrence of 'clear' sings of OA (= K-L scores of 2 or higher) in climbers at baseline and at 10 years follow-up, as well as in their age-matched controls for digit III. Data are expressed as joint and side-specific group relative proportion means with 95% CI. Non-overlapping 95% CIs between the groups were interpreted as significant differences at $p<0.05$; * climbers at baseline significantly differ from climbers at 10-years follow-up; # climbers at 10-years follow-up significantly differ from age matched controls.

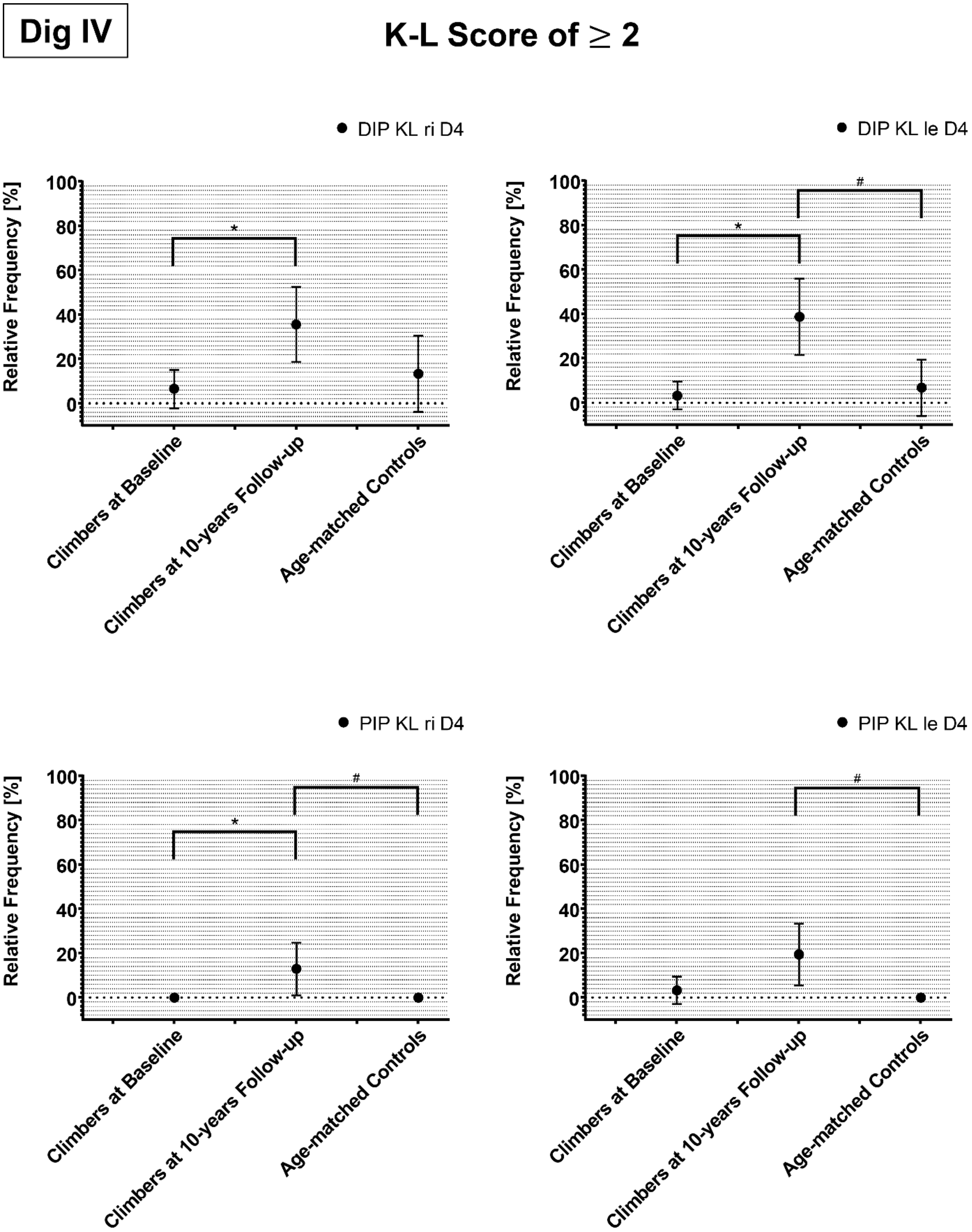

Supplemental Figure 2c: Occurrence of 'clear' sings of OA (= K-L scores of 2 or higher) in climbers at baseline and at 10 years follow-up, as well as in their age-matched controls for digit IV. Data are expressed as joint and side-specific group relative proportion means with 95% CI. Non-overlapping 95% CIs between the groups were interpreted as significant differences at $p<0.05$; * climbers at baseline significantly differ from climbers at 10-years follow-up; # climbers at 10-years follow-up significantly differ from age matched controls.

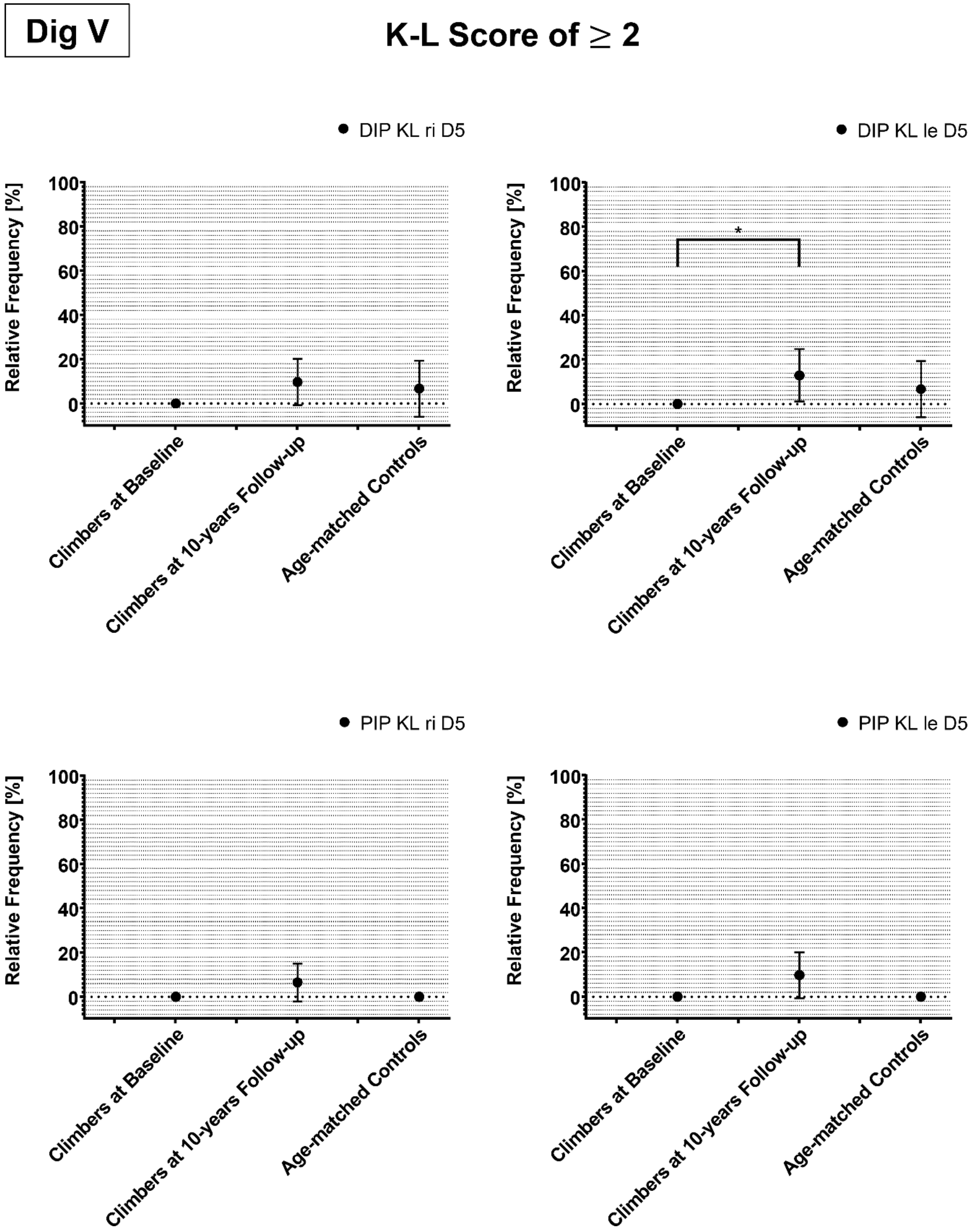

Supplemental Figure 2d: Occurrence of 'clear' sings of OA (= K-L scores of 2 or higher) in climbers at baseline and at 10 years follow-up, as well as in their age-matched controls for digit V. Data are expressed as joint and side-specific group relative proportion means with 95% CI. Non-overlapping 95% CIs between the groups were interpreted as significant differences at $p<0.05$; * climbers at baseline significantly differ from climbers at 10-years follow-up; # climbers at 10-years follow-up significantly differ from age matched controls.
